# Pharmacokinetic/Pharmacodynamic Relationships of Tulathromycin Against *Actinobacillus pleuropneumoniae* in a Porcine Tissue Cage Infection Model

**DOI:** 10.3389/fvets.2022.822432

**Published:** 2022-03-28

**Authors:** Lihua Yao, Lan Yang, Yuzhou Ling, Yanzhe Wei, Xiangguang Shen, Huanzhong Ding

**Affiliations:** Guangdong Key Laboratory for Veterinary Drug Development and Safety Evaluation, South China Agricultural University, Guangzhou, China

**Keywords:** tulathromycin, PK/PD relationship, *Actinobacillus pleuropneumoniae*, tissue cage infection model, %T > MIC

## Abstract

Tulathromycin is a semi-synthetic macrolide antibiotic that is highly effective in treating respiratory tract bacterial infections. We evaluated the *in vivo* antibacterial activity of tulathromycin against *Actinobacillus pleuropneumoniae* in piglets and determined its pharmacokinetic/pharmacodynamic (PK/PD) relationships using a tissue cage infection model. *A. pleuropneumoniae* (10^8^ CFU/ml) was exposed to tulathromycin via intramuscular injection followed by a collection of cage tissue fluids at various intervals. The percentage of time the drug concentration remained above the minimum inhibitory concentration (MIC) divided by the dosing interval (%T > MIC) was the best PK/PD index to describe the antibacterial efficacy of tulathromycin (*R*^2^ = 0.9421). The %T > MIC values required to achieve 1 – log_10_CFU/ml reductions and bactericidal activity (3 – log_10_CFU/ml reduction) were 50.8 and 96.38%, respectively. These results demonstrated that maintaining %T > MIC above 96.38% achieved bactericidal activity and thereby optimized the clinical dosage.

## Introduction

Porcine pleuropneumonia is a serious disease in pigs caused by *Actinobacillus pleuropneumoniae*, and its global dissemination has resulted in economic losses to the pork industry (1). *A. pleuropneumoniae* is a capsular Gram-negative bacterium (2) that is highly contagious and causes either acute or chronic fibro-hemorrhagic necrotizing pneumonia in pigs (3). Porcine pleuropneumonia can be complicated by the involvement of other pathogens such as pseudorabies virus, *Pasteurella multocida*, and *Mycoplasma pneumoniae* (4). The current treatments are vaccines (5, 6) and antibiotics including tilmicosin, ceftiofur, and enrofloxacin (7–9). However, excessive use of these drugs could lead to the development of antibiotic resistance (10). Therefore, it is particularly important to explore novel antibiotics or treatment strategies against *A. pleuropneumoniae* infections.

Tulathromycin is the first member of a novel subclass of macrolides known as triamilides (11) and is composed of two isomers that include 15- and 13-membered ring azalides at a ratio of 9:1 (12). It is a semi-synthetic macrolide containing three amine functional groups, is completely ionized at acidic conditions, and is positively charged (13). Tulathromycin can fully exert its bactericidal effect mainly due to it having better penetration of the molecule through the outer membrane (13). Tulathromycin possesses excellent pharmacokinetic properties with rapid absorption, high bioavailability, extensive tissue distribution, and long-lasting efficacy (12, 14). It has been approved by the US Food and Drug Administration (FDA) and European Medicines Agency (EMA) for use in the treatment and prevention of bovine respiratory disease associated with *Mannheimia haemolytica, A. pleuropneumoniae*, and *P. multocida* (15). Tulathromycin is effective against *A. pleuropneumoniae, P. multocida*, and *Mycoplasma hyopneumoniae* in swine (16).

Pharmacokinetic/pharmacodynamic (PK/PD) modeling is widely used to determine appropriate dosage regimens for antibacterial agents while decreasing side effects and bacterial resistance (17–20). To date, several models have been used for medical PK/PD synchronization including *in vitro, ex vivo*, and *in vivo* models (21–23). In the present study, we utilized an *in vivo* tissue cage infection model that reflects interactions between drug, host, and pathogen. A tissue cage model was first employed to explore the physiology and composition of interstitial fluid (24). Moreover, the tissue cage model has been applied to determine relationships between PK/PD parameters and antibacterial effectiveness in piglets (25, 26).

In the current study, a tissue cage infection model was adopted to determine the *in vivo* PK/PD parameters of tulathromycin against *A. pleuropneumoniae*. To the best of our knowledge, there is currently no such model used for tulathromycin against *A. pleuropneumoniae* infections. We therefore measured the *in vivo* antibacterial activity of tulathromycin against *A. pleuropneumoniae*, evaluated the relationship between PK/PD parameters and antibacterial effects, and then calculated the target values of the PK/PD parameter to achieve various antibacterial effects. Our results will provide an approach for guiding the rational use of tulathromycin in veterinary practice as well as provide a reference for minimizing the occurrence of resistant bacteria.

## Materials and Methods

### Organism, Antimicrobial Agent, and Chemicals

The *A. pleuropneumoniae* standard strain CVCC259 was obtained from the Chinese Veterinary Culture Collection Center (Beijing, China). Prior to testing, the organism was grown and subcultured at least three times on tryptic soy agar (TSA) and in tryptic soy broth (TSB) (Guangdong Huankai Microbial Science, Zhaoqing, China). TSB and TSA were supplemented with 4% newborn calf serum (Guangzhou Ruite Biotechnology, Guangzhou, China) and 1% nicotinamide adenine dinucleotide (NAD) (MYM Biological Technology, Beijing, China). Tulathromycin reference standard 99.8% was purchased from Shandong Lukang Shelile Pharmaceutical (Jining, China). Draxxin (20 ml: 2 g) was used as an injectable aqueous solution and was obtained from Fareva Amboise (Pocé sur Cisse, France). Pentobarbital sodium was provided by Jian Yang Biotechnology (Guangzhou, China). Procainamide hydrochloride was supplied by Xin Zheng, Tianjin Pharmaceutical (Tianjin, China).

### Animals

The animals used for this study were six castrated male and six female (Duroc × Landrace × Yorkshire) piglets weighing 14–16 kg and obtained from Guangzhou Fine Breed Swine Farm (Guangzhou, China) and were housed individually in crates for a week prior to experiments at a controlled temperature of 26°C. The animals received antibiotic-free fodder and water *ad libitum*. All the experimental procedures were approved and permitted by the Institutional Animal Care and Use Committee (IACUC) of South China Agricultural University (Approval Number: 2018A009).

### Construction of a Tissue Cage Infection Model

The tissue cage infection model was constructed as previously described (26). Briefly, piglets were anesthetized by pentobarbital, and local anesthesia was maintained by procainamide hydrochloride. Sterile tissue cages were implanted subcutaneously on the right and left sides of the neck of each anesthetized piglet. After surgery, all piglets were treated with antonidine injection for 3 to 5 days to relieve pain. Penicillin sodium was injected into piglets twice daily for 3 days to prevent infection. Tetracycline ointment was also applied topically at the wound sites. The surgical sites healed in 2–3 weeks, and the tissue cages were full of clear, yellow fluid. These fluids were collected from each tissue cage to confirm sterility and for minimum inhibitory concentration (MIC) determinations (see below). Approximately 1 week after collection, a 0.5-ml volume (at 10^8^ CFU/ml) of *A. pleuropneumoniae* was injected per cage in each animal and represented Day 0.

### Minimum Inhibitory Concentration Determination of Tulathromycin Against *Actinobacillus pleuropneumoniae*

The bacteria were grown as previously described (27) except that different media were used. Briefly, the bacterial were grown in supplemented TSB and TSA (see above). After incubation for 8 h in TSB in a constant-temperature shaker maintained at 37°C and 200 rpm/min, the logarithmic-phase bacteria were diluted to the desired concentration. After each dilution, measured with the UV spectrophotometer (OD_600nm_) until the reading is 0.2 (~5 × 10^5^ colony-forming units (CFU)/ml). Finally, the adjusted concentration of bacterial suspension was used to determine the MIC following the method of a prior publication (28). MICs in TSB, serum, and tissue cage fluids were determined as the lowest concentration that resulted in complete growth inhibition by visual assessment in triplicate, and mean values were used for data analysis.

### Experimental Design and Sample Collection

According to the results of the preliminary experiments, piglets were randomly divided into one control group and five treatment groups (two piglets, four tissue cages/group). For treatment groups, piglets received a single dose of tulathromycin (Draxxin) at 2.5, 5, 7.5, 10, and 20 mg/kg by intramuscular injection (IM). Controls were injected with sterile normal saline. Fluids were collected from the cages by percutaneous puncture at 0, 3, 6, 9, 12, 24, 48, 72, 96, 120, and 144 h after the tulathromycin dose. Fluid samples were clarified by centrifugation at 3,000 × *g* for 10 min, and 100 μl of the suspension was serially diluted 10-fold in saline for colony counting. The remaining samples were stored at −20°C until analysis by high-performance liquid chromatography–tandem mass spectrometry (HPLC-MS/MS) (see below).

### Time-Kill Curves *in vitro* and *in vivo*

*A. pleuropneumoniae* (10^8^ CFU/ml, with OD_600nm_ = 1.0) was inoculated into supplemented TSB containing tulathromycin at levels ranging from 1/4 to 32× MIC. The final concentrations of bacteria were 10^6^ CFU/ml. Cultures were incubated at 37°C for 12 h. Aliquots of 100 μl from each culture were removed at 0, 1, 2, 3, 4, 6, 9, and 12 h. Viable counts of an organism were determined via 10-fold serial dilutions in sterile physiological saline. Each diluted culture measuring 20 μl was spread on drug-free supplemented TSA agar plates and incubated at 37°C for 24 h for CFU determinations. A growth control consisting of *A. pleuropneumoniae* inoculum in the absence of tulathromycin was performed at the same time. The detection limit was 50 CFU/ml.

Following infection, fluids were collected from the tissue cages at 0, 3, 6, 9, 12, 24, 48, 72, 96, 120, and 144 h after each dose. The control samples were collected from piglets infected by *A. pleuropneumoniae* but not treated with tulathromycin. Once the samples were collected, they were used immediately for CFU determinations in triplicate.

### High-Performance Liquid Chromatography–Tandem Mass Spectrometry Method

Tissue cage fluids samples were naturally thawed, and 0.2 ml was added to a 1.5-ml centrifuge tube. The samples were deproteinized by adding an equal volume of acetonitrile followed by centrifugation at 13,000 × *g* for 10 min at 4°C. The supernatants (0.2 ml) were mixed with 0.8 ml HPLC mobile phase (0.1% formic acid:acetonitrile, 9:1) and filtered through a 0.22-μm nylon syringe filter for HPLC-MS/MS analysis. The limits of detection and quantification were 5 and 10 ng/ml, respectively.

### Data Analysis

Pharmacokinetic parameters of tulathromycin in tissue cage fluids were obtained using a two-compartment model using WinNonlin 5.2.1 software (Pharsight, Mountain View, CA, USA). The relationship between PK/PD parameters [the ratio of peak concentration divided by the MIC (C_max_/MIC) and the ratio of the area under the concentration–time curve divided by the MIC (AUC_0−24h_/MIC), %T > MIC)] and the *in vivo* antibacterial effect of tulathromycin were analyzed using the sigmoid E_max_ model provided by WinNonlin 5.2.1 software. The equation of this model was as follows:


$$\begin{array}{l} E=E_{0}+\left(E_{\max}-E_{0}\right)\times \frac{C_{e}^{N}}{EC_{50}^{N}+C_{e}^{N}} \end{array}$$

where E is the antibacterial effect measured as the change in log_10_CFU/ml in tissue cage fluids after 24-h incubation compared with the initial log_10_CFU/ml; E_0_ represents the change of bacterial load (log_10_CFU/ml) in the control group; E_max_ is maximum antibacterial effect for 24 h after drug administration; C_e_ stands for the PK/PD index magnitude, and N is the Hill coefficient that describes the sigmoid shape; EC_50_ represents corresponding PK/PD parameter value when the drug achieves one-half of the maximum antibacterial effect. According to this model, the correlation between the antibacterial effect and PK/PD parameters can be reflected through the *R*^2^ value.

## Results

### Determination of the Minimum Inhibitory Concentration

The MIC values of tulathromycin against *A. pleuropneumoniae* CVCC259 were 1, 0.25, and 0.25 μg/ml in supplemented TSB, serum, and tissue cage fluid, respectively. The MIC values determined in TSB were 4-fold higher than for the 2 biological samples.

### *In vitro* Time-Killing Studies

**Figure 1:**
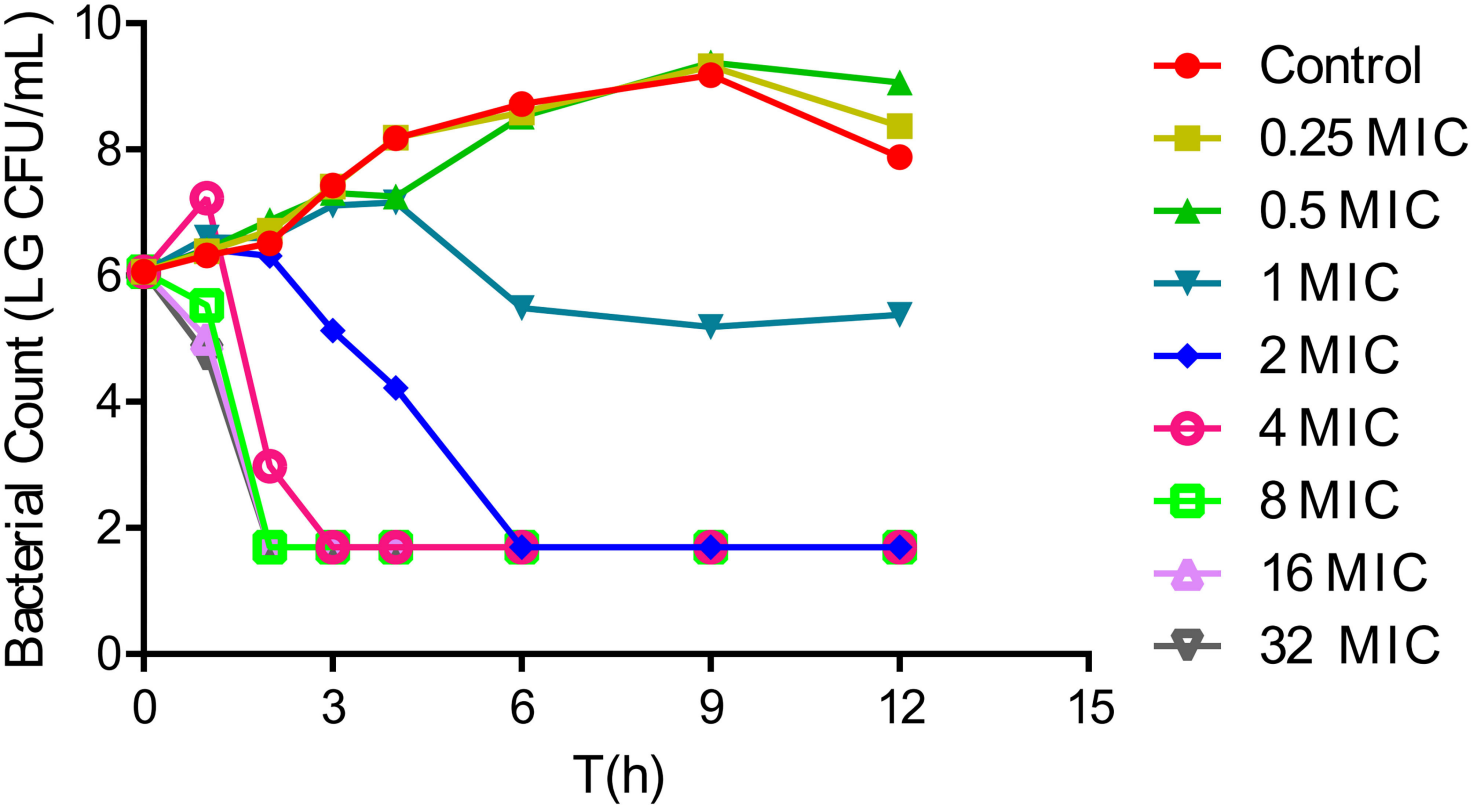
*In vitro* time-kill curves for tulathromycin against *Actinobacillus pleuropneumoniae* CVCC259 in tryptic soy broth (TSB) (containing 1% NAD (1 mg/ml) and 4% newborn calf serum).

*In vitro* time-killing curves were used to describe the antibacterial activity of tulathromycin in the range of 0.25–32 × MIC multiples against *A. pleuropneumoniae* CVCC259. Tulathromycin at concentrations of 0.25× and 0.5× MIC did not appreciably inhibit bacterial growth. However, exposure to 1× MIC resulted in the gradual decline of CFU from 4 h. Moreover, 2× MIC resulted in large reductions in CFU values, and no visible colonies were apparent after 6 h. Tulathromycin at 4–32 × MIC did not achieve a more rapid or greater reduction in CFU, indicating that the bactericidal activity was not dependent on concentration but rather time. Interestingly, there were rapid and large CFU reductions of 4 – log_10_CFU/ml at tulathromycin levels of 4–32× MIC (Figure 1).

### *In vivo* Antibacterial Studies of Tulathromycin

**Figure 2:**
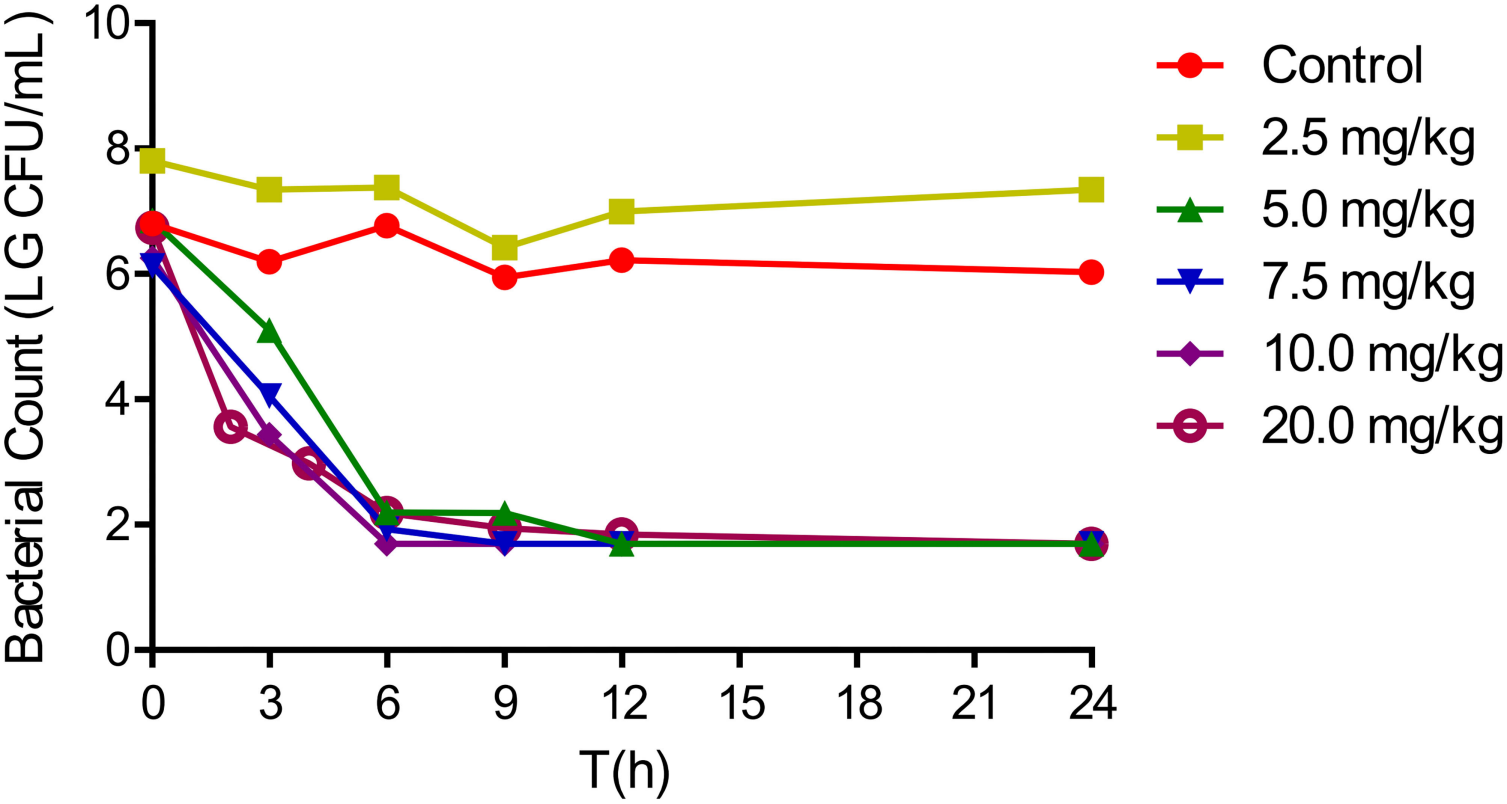
*In vivo* antibacterial activity of tulathromycin against *Actinobacillus pleuropneumoniae* CVCC259 at different concentrations.

The *in vivo* antibacterial effect of tulathromycin against *A. pleuropneumoniae* CVCC259 in tissue cage fluids was determined after the animals were administered with different tulathromycin doses. In fluids collected up to 144 h, tulathromycin exerted a notable bactericidal effect, while bacterial counts for the blank control group were maintained at about 10^7^ CFU/ml. However, no inhibition of bacterial growth was observed for tissue cage fluids harvested when tulathromycin was administered at 2.5 mg/kg. The administration of 5 mg/kg resulted in a 5 – log_10_CFU/ml reduction in bacteria within 9 h, and no bacteria were detected after 12 h. Tulathromycin at doses of 5 mg/kg and above had a strong bactericidal activity that resulted in at least a 4 – log_10_CFU/ml reduction (Figure 2).

### Relationships Between Pharmacokinetic/Pharmacodynamic Parameters and Effectiveness

**Figure 3:**
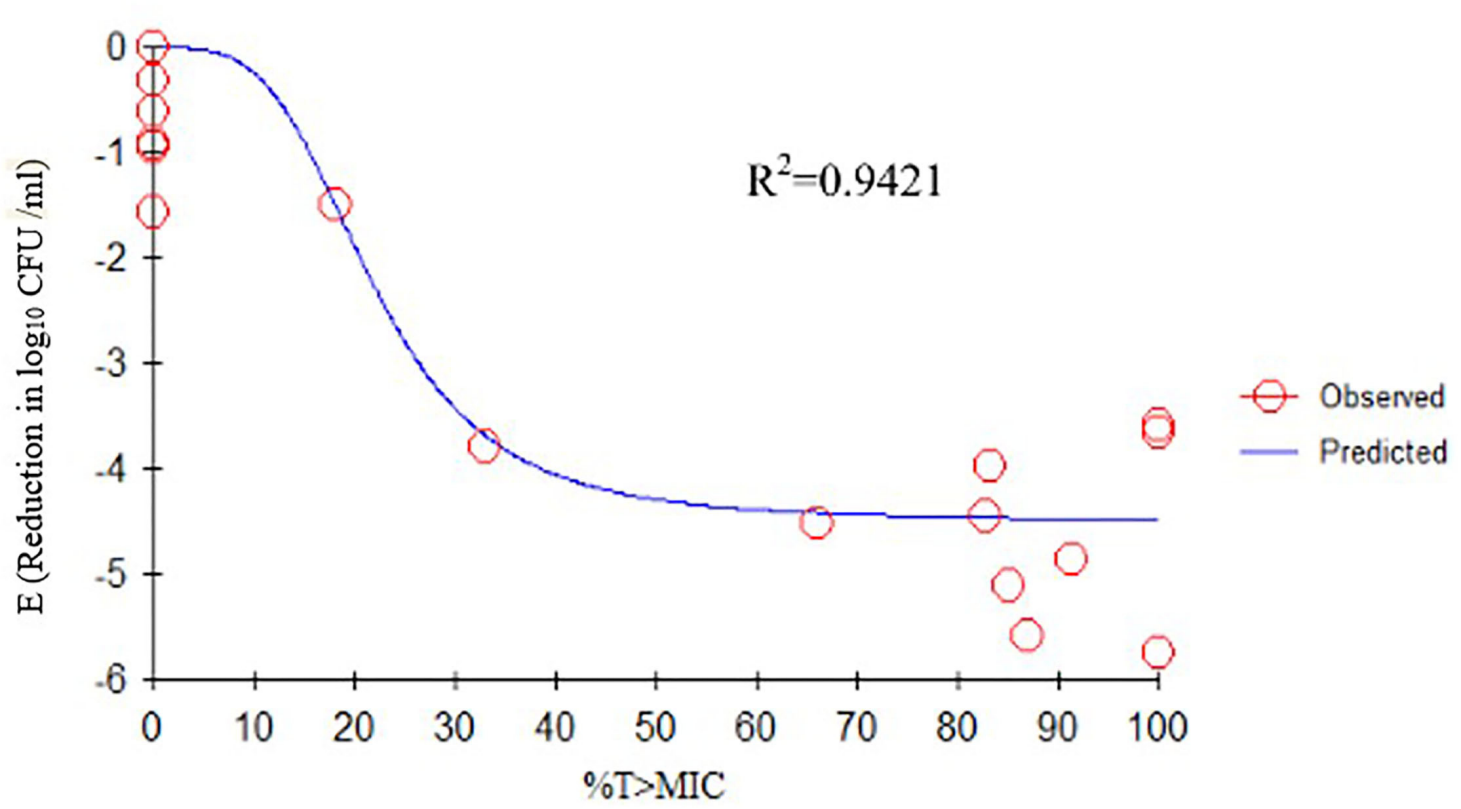
E_max_ relationships between PK/PD parameters (%T > MIC) and antibacterial activity. *R*^2^ is the correlation coefficient. PK/PD, pharmacokinetic/pharmacodynamic; MIC, minimum inhibitory concentration.

We determined the relationships between the antibacterial effectiveness of tulathromycin against *A. pleuropneumoniae* and the PK/PD indices AUC_0−24*h*_/MIC, C_max_/MIC, and %T > MIC. The effects of tulathromycin against *A. pleuropneumoniae* were highly correlated with %T > MIC (*R*^2^ = 0.9421) and AUC_0−24h_/MIC (*R*^2^ = 0.9391). However, by combining *in vitro* and *in vivo* time-killing studies, we confirmed that %T > MIC was a more appropriate PK/PD parameter to describe the antibacterial effect of tulathromycin against *A. pleuropneumoniae* (Figure 3). These results were similar to the *in vitro* time-killing study and indicated that tulathromycin was a time-dependent antibacterial agent. Additionally, C_max_/MIC and AUC_0−24h_/MIC indices also had a high value of *R*^2^ (C_max_/MIC, *R*^2^ = 0.8676; AUC_0−24h_/MIC, *R*^2^ = 0.9391). Therefore, the antibacterial effectiveness of tulathromycin may also correlate with concentration.

### *In vivo* Pharmacokinetic/Pharmacodynamic Integration and Modeling

The tissue cage fluid pharmacokinetic parameters of tulathromycin following a single-dose IM (Table 1) were used in conjunction with cage fluid MIC data and used for PK/PD integration and modeling. A sigmoid E_max_ model was adopted to integrate and simulate the relationship between the effect of tulathromycin against *A. pleuropneumoniae* and the PK/PD parameters in the tissue cage infection model. The calculated %T > MIC values required to produce 1 – log_10_CFU/ml and 3 – log_10_CFU/ml killing activities were 50.8% and 96.38%, respectively. The slope of the curve of the %T > MIC versus antibacterial activity was 3.671 (Table 2). Results on the PK/PD of tulathromycin against *A. pleuropneumoniae* in the tissue cage infection model were depicted in Table 3.

**Table 1:** Tissue cage fluid pharmacokinetic parameters of tulathromycin following a single-dose intramuscular injection.

**Dosage (mg/kg)**	**AUC_0−24*h*_ (μg·h/ml)**	**C_max_ (μg/ml)**
2.5	0.561 ± 0.138	0.037 ± 0.002
5	3.542 ± 0.433	0.317 ± 0.035
7.5	7.343 ± 0.708	0.408 ± 0.010
10	8.295 ± 2.340	0.568 ± 0.066
20	17.058 ± 1.805	1.011 ± 0.096

*AUC_0-24h_ was calculated by the non-compartment model of the WinNonlin software; C_max_ was the maximum concentration observed in tissue cage fluids within 24 h. The values shown are arithmetic means ± SD (n = 4)*.

**Table 2:** The values of PK/PD parameters and the %T > MIC values required for the indicated antibacterial effects.

E_max_ (log_10_CFU/ml)	−5.74
EC_50_ (h)	100
E_0_ (log_10_CFU/ml)	−0.6072
Slope (N)	3.671
%T > MIC for 1 – log_10_CFU/ml drop	50.80
%T > MIC for bactericidal effect	96.38

*E_max_, the maximum antibacterial effect within 24-h treatment period; EC_50_, the %T > MIC value produces a 50% reduction of the maximum antibacterial effect; E_0_, the change of the bacterial count (log_10_CFU/ml) in the control samples during 24 h; N, the Hill coefficient; PK/PD, pharmacokinetic/pharmacodynamic*.

**Table 3:** Study on the PK/PD of tulathromycin against *Actinobacillus pleuropneumoniae* in the tissue cage infection model.

**ΔE**	**AUC_**0−24**_/MIC**	**C_**max**_/MIC**	**T > MIC**
**(LgCFU/mL)**	**(h)**		**(%)**
−1.57403	0	0	0
−0.00976	0	0	0
−0.6072	0	0	0
−0.91285	0	0	0
−0.32451	1.69488	0.142	0
−0.96614	2.7966	0.1572	0
−4.85126	15.8988	1.1256	–
−2.52904	–	–	9.9827
−1.49049	–	1.408	18.0864
−4.51851	25.368	1.668	66.1074
−5.12057	31.524	1.576	85.1744
−3.65321	31.224	1.648	100
−3.57978	43.68247	2.611288	100
−4.45593	34.9076	2.237736	82.8146
−3.78533	20.95205	1.96784	33.1407
−4.96379	68.8832	3.452	93.8357
−4.44716	86.714	4.48	95.6677
−4.0569	67.468	4.56	92.4625
−3.94448	59.6616	3.78	72.687
−4.86332	58.2086	3.712	91.4419
−5.57978	78.628	4.68	87.1528
−5.74036	68.094	3.78	100
−3.98227	67.992	4	81.3218

*ΔE is the antibacterial effect measured as the change in Lg CFU/ml in TCF after 24 h incubation compared with the initial Lg CFU/ml, “–” indicates no data*.

## Discussion

Tulathromycin is a semi-synthetic macrolide and has the potential as an antibacterial agent against swine and cattle respiratory diseases caused by *A. pleuropneumoniae* and *P. multocida* (13). Its efficacy is characterized by a rapid rate of absorption, high bioavailability, extensive tissue distribution, and longer elimination half-lives in the plasma and lungs of pigs (29, 30). In the current work, we confirmed that tulathromycin possessed a bactericidal activity against *A. pleuropneumoniae* both *in vitro* and *in vivo*. *In vitro* time-killing curves demonstrated that a reduction in *A. pleuropneumoniae* counts to the detection limit was achieved at concentrations of 2 × MIC and greater within 6 h (Figure 1). Additionally, after a single IM dose of 5 mg/kg, a 5 – log_10_CFU/ml reduction could be observed within 9 h, and no bacteria were detected after 12 h (Figure 2). Moreover, the antibacterial activity of tulathromycin against *A. pleuropneumoniae* was time-dependent, and longer drug exposure and bacterial killing were positively correlated. Furthermore, the curve of 8–32 × MIC was coincident and indicated that increased drug levels had no significant effect on bactericidal activity. Similar results have been previously reported for tulathromycin against *P. multocida* (31, 32). Tulathromycin exhibits strong bactericidal activity against *A. pleuropneumoniae*, and this has been previously reported (33).

To date, *ex vivo* PK/PD studies for tulathromycin have been conducted using *P. multocida, Streptococcus suis*, and *Glaesserella parasuis* in pigs (31, 34, 35) and against *M. haemolytica* and *P. multocida* in cattle (36). All those studies explored the efficacy of tulathromycin against different pathogens, and the PK/PD parameter that best described the antibacterial activity was AUC_0−24h_/MIC. However, *in vivo* PK/PDs for tulathromycin against *A. pleuropneumoniae* had not been determined. Therefore, we initially established a tissue cage infection model *in vivo* to explore the efficacy and PK/PD relationships of tulathromycin against *A. pleuropneumoniae*.

Generally, the *in vivo* model is more reliable and applicable than *ex vivo* and *in vitro* models because this model acquires data relating to the dynamic interaction of multiple factors associated with the host, antibacterial agent, and pathogen. However, *ex vivo* and *in vitro* models both ignore the natural rates of body clearance in the drug metabolism of the animal.

The findings from this study showed that correlation values of %T > MIC (*R*^2^ = 0.9421) and AUC_0−24h_/MIC (*R*^2^ = 0.9391) were very close, which was perplexing, as the bactericidal action of macrolides is not always predictable. For example, erythromycin was considered a time-dependent macrolide antibiotic, while clarithromycin and azithromycin were concentration-dependent (37). In the current study, the %T > MIC role was evaluated by integrating the *in vitro* and *in vivo* time-killing studies.

**Figure 4:**
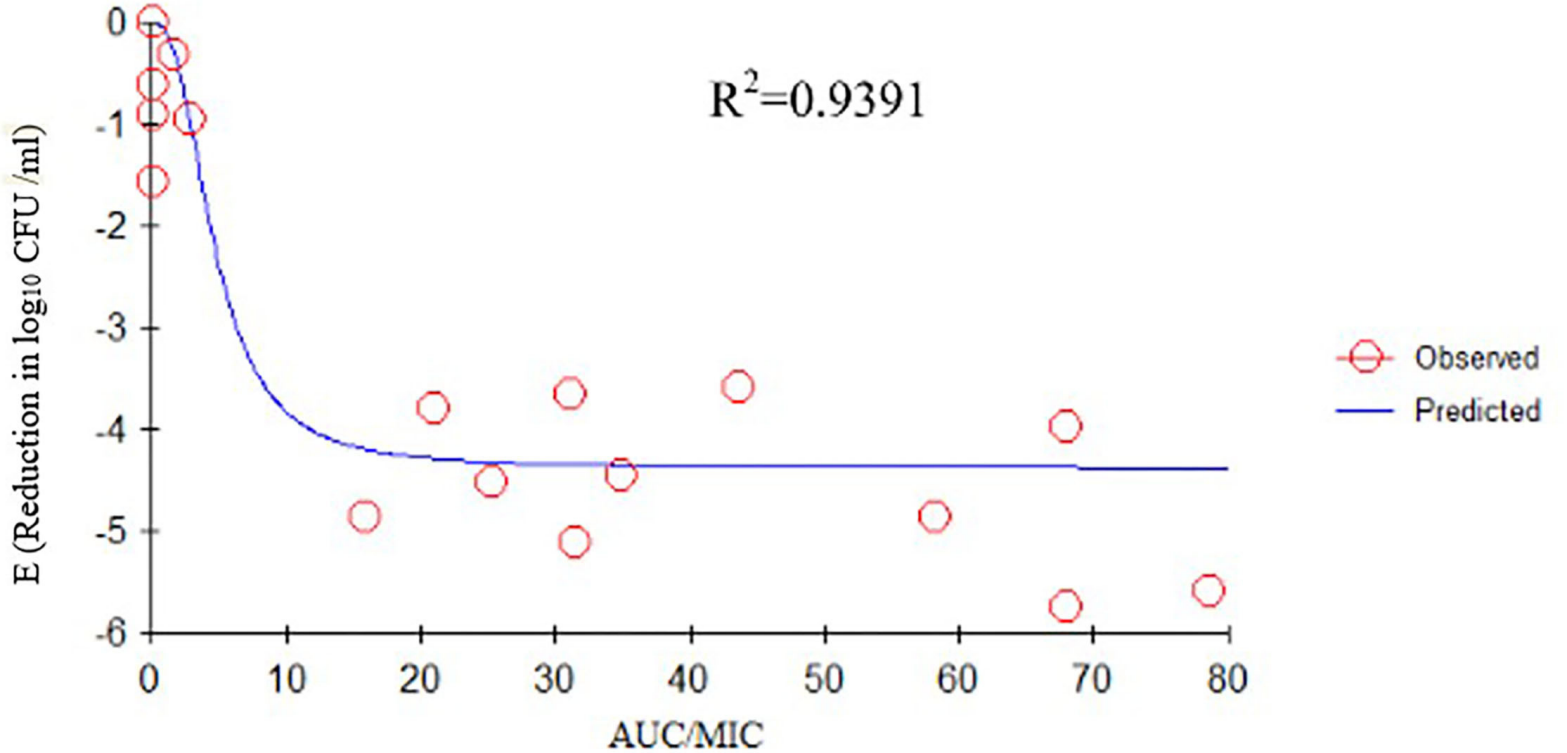
E_max_ relationships between PK/PD parameters (AUC_0−24h_/MIC) and antibacterial activity. *R*^2^ is the correlation coefficient. PK/PD, pharmacokinetic/pharmacodynamic; MIC, minimum inhibitory concentration.

**Figure 5:**
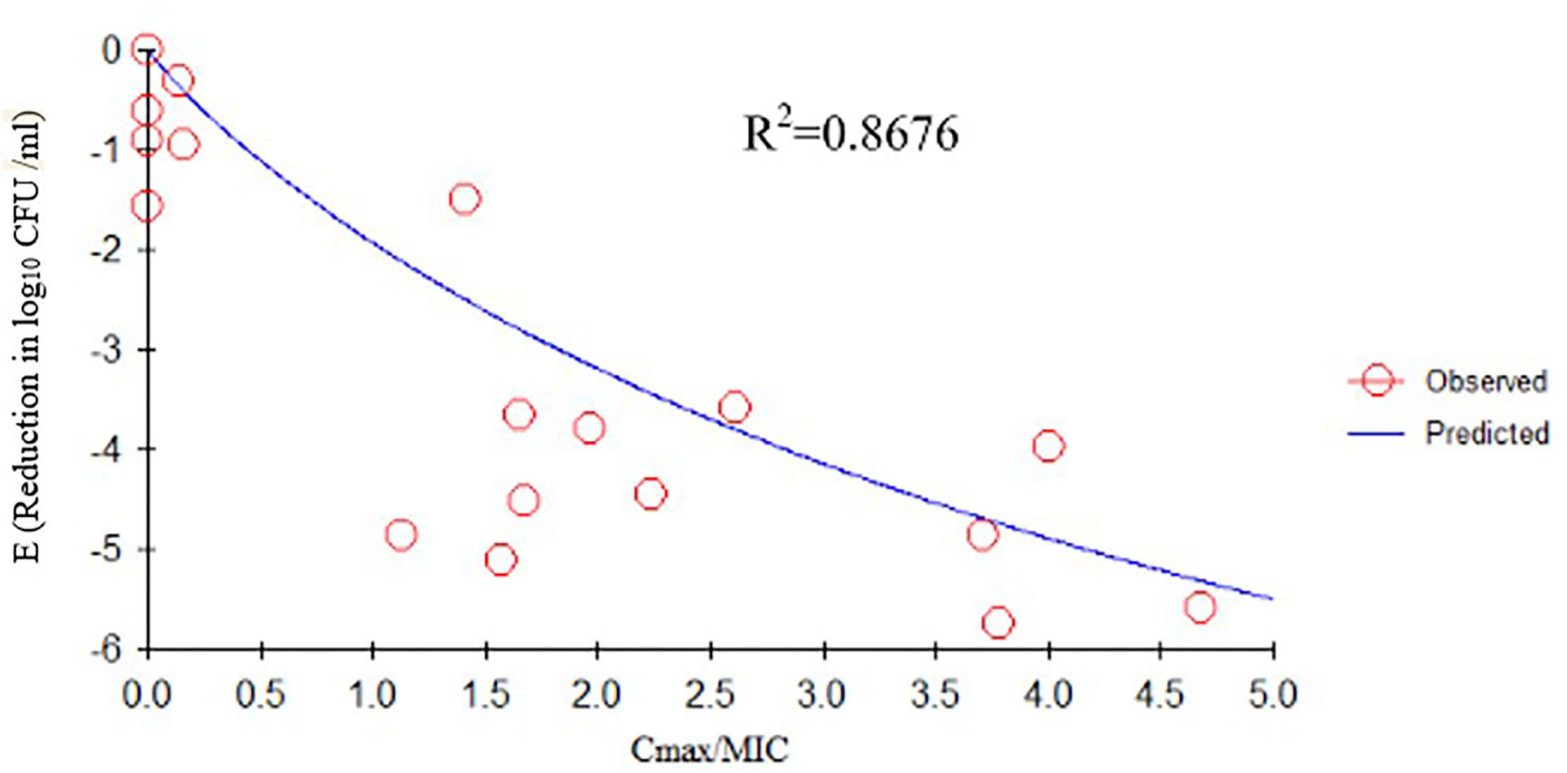
E_max_ relationships between PK/PD parameters (C_max_/MIC) and antibacterial activity. *R*^2^ is the correlation coefficient. PK/PD, pharmacokinetic/pharmacodynamic; MIC, minimum inhibitory concentration.

Previous studies have indicated differences in tulathromycin activity against different bacterial species. For instance, tulathromycin was considered a bacteriostatic agent against *Staphylococcus aureus* and *Escherichia coli* (38) but a bactericidal against *M. haemolytica, A. pleuropneumoniae*, and *P*. *multocida* at 4× and 8× MIC (13, 33). In the present study, our data demonstrated a good correlation between %T > MIC, AUC_0−24h_/MIC, and C_max_/MIC, and the antibacterial effects were 0.9421, 0.9391, and 0.8676, respectively (Figures 3–5). These data indicated that %T > MIC was the most appropriate PK/PD index to predict the antibacterial activity of tulathromycin against *A. pleuropneumoniae*.

Tulathromycin possessed a characteristic of time dependence against *A. pleuropneumoniae* and PK/PD-based modeling with the sigmoid E_max_ model indicating that animal dose regimens should supply %T > MIC of tulathromycin at least 50.8% to achieve a 1 – log_10_CFU/ml reduction and 96.38% to achieve a bactericidal effect. We also found that the antibacterial effect was favorably correlated to the time that the drug concentration exceeded MIC and this differed from previous studies (31, 35). The reasons for these differences are severalfold; *ex vivo* and *in vivo* models possess differences, the *ex vivo* model holds the antibiotic concentration constant, and the response was independent of host immunity. In addition, pathogen differences and bacterial loads also influence the magnitude of PK/PD parameters. A higher antimicrobial concentration would be required for higher pathogen loads (39, 40).

Although we obtained some useful *in vivo* PK/PD data about tulathromycin against *A. pleuropneumoniae* by establishing a tissue cage infection model, there are some limitations of this model. Firstly, the lung and not the tissue cage was the site of the infection. Although tissue cage fluids represent a deep peripheral compartment, they are not identical to interstitial fluid. Secondly, as far as we know, tulathromycin accumulates in lung tissue or intra-airway compartments (12) so that levels in tissue cage fluids would differ from pulmonary epithelial lining fluid (PELF) or bronchial epithelial lining fluid (BELF). Therefore, a lung infection model would be more appropriate to better explore the *in vivo* PK/PD integration of tulathromycin against *A. pleuropneumoniae*. We plan to establish a lung infection model or identify concentration conversion coefficients between tissue cage fluids and lung tissues in our future studies.

## Conclusion

In this study, we established a porcine tissue cage infection model to evaluate for the first time the *in vivo* antibacterial activity of tulathromycin against *A. pleuropneumoniae* and explored PK/PD relationships. Our data demonstrated that tulathromycin exhibited a time-dependent killing efficacy against *A. pleuropneumoniae* and %T > MIC (*R*^2^ = 0.9421) was the best PK/PD index to describe the antibacterial activity. Based on the PK and PD data, further analysis of the results showed that the values of %T > MIC for tulathromycin required 1 – log_10_CFU/ml reduction, and bactericidal effects in tissue cage fluids were 50.8 and 96.38%. These results demonstrated a 99.9% bacterial killing when the value of %T > MIC was 96.38. Our results would be helpful and meaningful to guide and establish PK/PD cutoffs for this drug. Future studies are planned to further integrate our present PK/PD findings and MIC distributions of *A. pleuropneumoniae* strain in China to design targets and optimize the clinical dosing regimens for treating *A. pleuropneumoniae* infections in piglets.

## Data Availability Statement

The original contributions presented in the study are included in the article/supplementary material, further inquiries can be directed to the corresponding author/s.

## Ethics Statement

The animal study was reviewed and approved by Institutional Animal Care and Use Committee (IACUC) of South China Agricultural University.

## Author Contributions

HD conceived this experiment and participated in its design and coordination. LYao designed and conducted this experiment and drafted the manuscript. LYang and YL performed the experimental procedure and participated in the PK/PD data analysis. LYang, YL, and YW collected and analyzed the samples. XS and HD revised the manuscript and supervised the entire manuscript. XS provided technical help about HPLC-MS/MS. All authors read and approved the final manuscript.

## Funding

This work was supported by Local Innovative and Research Teams Project of Guangdong Pearl River Talents Program (2019BT02N054).

## Conflict of Interest

The authors declare that the research was conducted in the absence of any commercial or financial relationships that could be construed as a potential conflict of interest.

## Publisher's Note

All claims expressed in this article are solely those of the authors and do not necessarily represent those of their affiliated organizations, or those of the publisher, the editors and the reviewers. Any product that may be evaluated in this article, or claim that may be made by its manufacturer, is not guaranteed or endorsed by the publisher.
